# Multidisciplinary Digital Therapeutics for Chronic Low Back Pain Versus In-Person Therapeutic Exercise with Education: A Randomized Controlled Pilot Study

**DOI:** 10.3390/jcm13237377

**Published:** 2024-12-04

**Authors:** Dong-Ho Kang, Jae Hyeon Park, Chan Yoon, Chi-Hyun Choi, Sanghee Lee, Tae Hyun Park, Sam Yeol Chang, Seong-Ho Jang

**Affiliations:** 1 Department of Orthopedic Surgery, Samsung Medical Center, Seoul 06351, Republic of Korea; 2 Department of Orthopedic Surgery, Seoul National University College of Medicine, Seoul 03080, Republic of Korea; 3 Department of Rehabilitation Medicine, Hanyang University College of Medicine, Guri-si 11923, Republic of Korea; jhpark3.md@gmail.com; 4 EverEx, Seoul 06641, Republic of Korea; chan.yoon@everex.co.kr (C.Y.); leo@everex.co.kr (C.-H.C.); ellie@everex.co.kr (S.L.); bill@everex.co.kr (T.H.P.); 5 Department of Orthopedic Surgery, Seoul National University Hospital, Seoul 03080, Republic of Korea

**Keywords:** chronic low back pain, digital therapeutics, cognitive behavioral therapy, exercise therapy, multidisciplinary treatment, randomized controlled trial

## Abstract

**Background:** Chronic lower back pain (CLBP) is a global health issue leading to significant disability and socioeconomic burden. Traditional treatments, including exercise and cognitive behavioral therapy (CBT), are often limited by physical and temporal constraints. This study aimed to evaluate the efficacy of multidisciplinary digital therapeutics (MORA Cure LBP) compared to conventional treatments. **Methods:** This multicenter, randomized, controlled pilot study enrolled 46 participants. Participants were randomly assigned in a 1:1 ratio to either a MORA Cure LBP group or control group, which received conventional treatment. **Results:** At eight weeks, both groups demonstrated improvements compared to baseline. No statistically significant differences were observed between the MORA Cure LBP and control groups in reductions in usual pain intensity (MORA Cure LBP: 3.1 ± 1.9 vs. control: 3.0 ± 1.5, *p* = 0.809), worst pain intensity (MORA Cure LBP: 5.00 ± 2.18 vs. control: 4.27 ± 1.83, *p* = 0.247), and functional disability (ODI, MORA Cure LBP: 15.6 ± 9.6 vs. control: 15.6 ± 10.0, *p* > 0.999). Compliance was significantly higher in the MORA Cure LBP group during the first 4 weeks (MORA Cure LBP: 74.7% ± 27.4 vs. control: 53.1% ± 28.6, *p* < 0.001). **Conclusions:** Both multidisciplinary digital therapeutics (MORA Cure LBP) and conventional treatments were effective in reducing pain and functional disability in patients with CLBP, with no significant differences between the two groups. Digital therapeutics, particularly those that integrate CBT and exercise, offer promising alternatives to conventional therapies by improving accessibility and potentially enhancing patient engagement.

## 1. Introduction

Chronic low back pain (CLBP) is a significant global health concern, with a lifetime prevalence exceeding 70% in industrialized countries and worldwide prevalence of 84% [1,2]. It imposes a substantial socioeconomic burden [3,4] and is the leading cause of disability worldwide [5]. Although acute low back pain often resolves within 4–6 weeks [6], chronic cases have a poorer prognosis, with up to 65% of patients experiencing persistent pain for 12 months or longer [7]. Several biological, psychological, social, and occupational factors are associated with poor clinical outcomes in patients with CLBP. These factors include severe disability, sciatica, advanced age, poor overall health, increased psychological or psychosocial distress, negative cognitive characteristics, poor relationships with colleagues, excessive physical labor demands, and secondary compensation [8]. Although many clinical guidelines have focused on pharmacological treatments, physical therapy, and appropriate management of patients with radicular pain [9], a recent study suggests that moderate-to-severe CLBP is associated with reduced health-related quality of life, health status, increased absenteeism, and increased healthcare utilization, regardless of whether patients use prescription medications [10,11].

For chronic low back pain, pharmacological interventions alone are limited to short-term benefits (<3 months) and are associated with increased adverse effects compared to placebo, according to a recent meta-analysis [12]. Therefore, emerging clinical guidelines recommend a multidisciplinary approach or collective back pain treatment that combines pharmacological interventions with psychological interventions, exercise therapy, and invasive treatments [9,11,13,14]. Among psychological interventions, cognitive behavioral therapy (CBT) has shown well-established effectiveness in improving CLBP outcomes [15,16,17,18,19]. A recent meta-analysis reported that these psychological interventions are more effective for pain relief and physical function enhancement when provided in combination with physical or exercise therapy rather than as standalone treatments [20]. However, traditional face-to-face CBT and physical or exercise therapy face challenges related to physical and time constraints, potentially leading to low adherence to such collective back pain treatments. Therefore, to improve treatment effectiveness, there is a need to increase the accessibility of cognitive–behavioral and exercise therapies, which can be achieved through the development of digital therapeutic devices utilizing artificial intelligence (AI), portable devices, and applications.

Based on this background, we developed the MORA Cure LBP, which is a digital therapeutic device for managing and treating CLBP. This study aimed to evaluate the efficacy of MORA Cure LBP compared to conventional therapy, including in-person therapeutic exercise with education in patients with CLBP. We hypothesized that the MORA Cure LBP group would show greater improvements in pain intensity, overall treatment effect, lumbar function, quality of life, muscle strength, and psychological state than the in-person therapeutic exercise with education group. The primary outcomes of this randomized controlled pilot study were usual pain intensity, worst pain intensity, and functional disability (as measured using the Oswestry Disability Index [ODI]) at eight weeks post-baseline. In this study, we assessed the potential of MORA Cure LBP as an accessible and effective digital therapeutic option for CLBP management, potentially addressing the limitations of traditional face-to-face therapies and improving treatment adherence.

## 2. Materials and Methods

### 2.1. Study Design and Ethical Considerations

This study was a multicenter, prospective, randomized controlled trial conducted at the Seoul National University Hospital and Hanyang University Guri Hospital. This trial aimed to evaluate the efficacy of multidisciplinary digital therapeutics (MORA Cure LBP) compared to conventional treatment, which includes in-person therapeutic exercise with education for CLBP. The study protocol was approved by the Institutional Review Boards of Seoul National University Hospital (IRB number: 2211-148-1381) and Hanyang University Guri Hospital (IRB number: 2022-12-049). All procedures were conducted in accordance with the ethical standards of the responsible committees on human experimentation (institutional and national) and the Declaration of Helsinki of 1975, as revised in 2000.

### 2.2. Participants

Eligibility criteria included adults aged 18–65 years with CLBP persisting for >12 weeks and a minimum average pain score of 3 on the 11-point Numeric Pain Rating Scale (NPRS). The participants were required to be capable of using a smartphone application for treatment and provide voluntary informed consent. Exclusion criteria included any history of previous spinal surgery, spinal injection therapy within one month prior to enrollment, or spinal trauma within three months. Participants were also excluded if they presented with radicular pain with sensory or motor deficits, leg muscle strength of grade ≤3 on manual muscle testing, or structural spinal abnormalities, such as spondylolisthesis or scoliosis with a Cobb angle >10°. Those exhibiting red flag signs, such as unexplained weight loss or bowel/bladder dysfunction, or those with tumors, infections, metabolic bone diseases, cognitive disorders, fibromyalgia, or systemic inflammatory diseases, were also excluded. Further exclusion criteria included pregnancy or breastfeeding, current use of opioids stronger than tramadol, a history of substance abuse or psychiatric conditions affecting pain perception, and an inability to communicate or follow instructions.

### 2.3. Randomization and Blinding

The participants were randomly assigned in a 1:1 ratio to either a DTx group (MORA Cure LBP digital therapeutics) or a control group (conventional treatment) using a stratified randomization method. Stratification was based on the study site, Seoul National University Hospital or Hanyang University Guri Hospital. Randomization was performed using a web-based program provided by the Seoul National University Medical Research Collaboration Center. This study was open-label; however, allocation concealment was maintained by independent study staff who were not involved in the data collection or analysis.

### 2.4. Interventions

**Figure 1:**
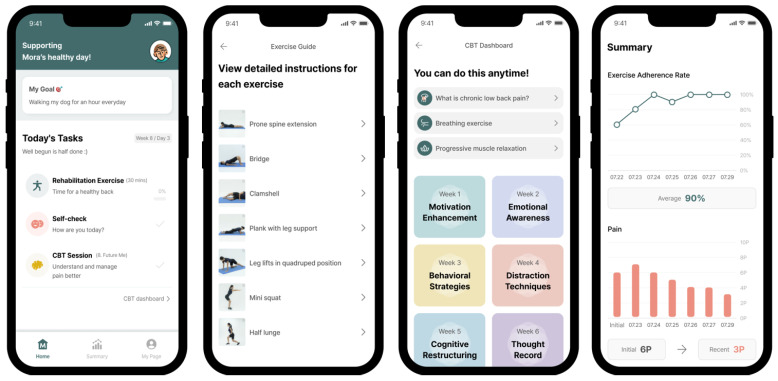
Example of the patient user interface of MORA Cure LBP. After pose estimation, real-time feedback was provided to assist patients in adopting correct postures during exercise sessions. Additionally, CBT sessions and worksheet records are displayed for patient reference and progress tracking.

Participants in the DTx group underwent an eight-week program using the MORACure LBP digital therapeutic device, which included a smartphone application delivering CBT and exercise therapy (Figure 1). The program comprised one CBT session at the beginning of each week, followed by daily exercise sessions for the remaining six days. These CBT sessions addressed the identification and management of negative emotions and thoughts associated with pain, fostering positive coping skills and applying relaxation techniques. The program was designed to enhance the participants’ pain management self-efficacy through cognitive restructuring, positive self-talk, and strategies for handling setbacks. Patients underwent patient-tailored exercise therapy, education, and behavioral therapy. Evaluation using AI pose estimation and the results of subjective symptom evaluation were used to create a patient-tailored treatment curriculum. The exercises focused on core and lower extremity strengthening and incorporated stretching, with adjustments based on pain scales, following the principles of progressive overload. Participants in the control group received conventional treatment, including exercise therapy with controlled exercise intensity, which included up to four face-to-face sessions with a physician experienced in musculoskeletal disorders. They also engaged in self-directed exercises based on educational materials provided at baseline, with a treatment duration of eight weeks, mirroring that of the DTx group. Additionally, the last exercise therapy session was repeated for the remaining four weeks after the eight-week course.

### 2.5. Outcome Measures and Data Collection

The primary outcomes of this study included changes in usual pain intensity, as assessed by the NPRS, at baseline and after eight weeks. Additionally, the worst pain intensity, measured using the NPRS at these time points, and functional disability, assessed using the ODI [21], constituted the primary outcomes. Secondary outcomes included health-related quality of life assessed using the EQ-5D at baseline and at 4, 8, and 12 weeks [22]. Psychological status was measured using the Patient Health Questionnaire-9 (PHQ-9) [23], Pain Catastrophizing Scale (PCS) [24], and Fear-Avoidance Beliefs Questionnaire (FABQ) at baseline and 8 and 12 weeks [25]. Higher ODI scores indicate greater levels of functional disability, while higher NPRS scores reflect more intense pain. Conversely, lower EQ-5D scores represent poorer health-related quality of life. For psychological measures, higher PHQ-9, PCS, and FABQ scores denote greater levels of depression, pain catastrophizing, and fear-avoidance beliefs, respectively. Spinal alignment was evaluated using radiographic assessment at baseline and eight weeks, while muscle endurance and balance were assessed using the Prone Bridge Test and Single-Limb Stance Test at baseline and 4, 8, and 12 weeks [26,27]. Data were collected at baseline and at 4, 8, and 12 weeks (four weeks post-intervention) through a combination of in-person visits and telephone follow-ups. The participants were instructed to refrain from using rescue medication for 24 h prior to the assessment visits to minimize its effect on the outcome measures. Treatment compliance was assessed at 4, 8, and 12 weeks after the baseline. Compliance was calculated as the percentage of sessions completed relative to the total number of prescribed sessions. The DTx group automatically collected compliance data as time data from the application, whereas the control group wrote their own logs. Comparisons between the intervention and control groups were made at each time point to evaluate adherence to the treatment protocol.

### 2.6. Statistical Analysis

Continuous variables are summarized as mean ± standard deviation, while categorical variables are presented as frequencies and percentages. Between-group comparisons were conducted using two-sample *t*-tests for continuous variables and the Pearson’s chi-squared tests for categorical variables. Wilcoxon’s rank-sum tests were performed for non-normally distributed data. Primary outcomes were analyzed using repeated-measures analysis of variance, and changes over time within each group were assessed using paired *t*-tests. All statistical analyses were performed using SAS software (version 9.4), with the significance level set at *p* < 0.05.

## 3. Results

### 3.1. Participant Characteristics

**Figure 2:**
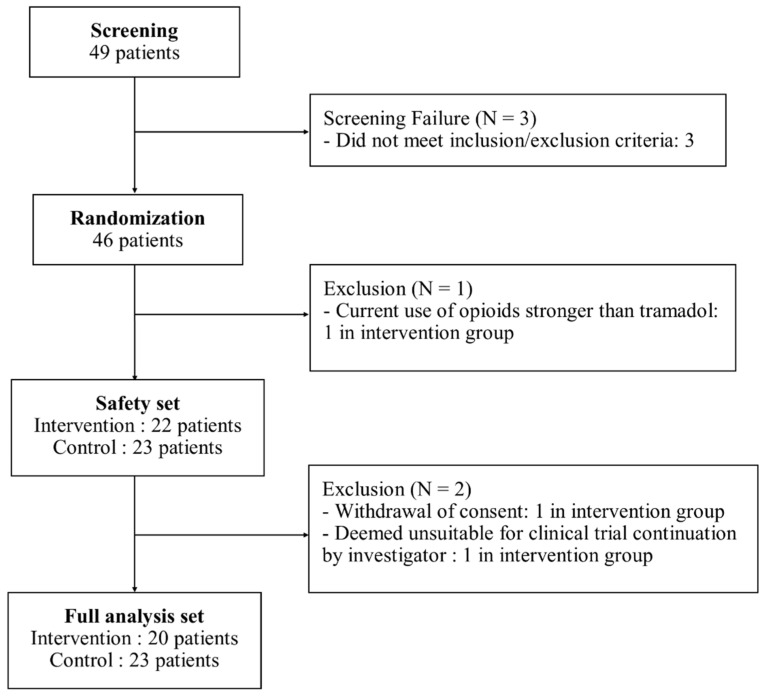
Flowchart of participants.

In total, 46 participants were randomized, with 45 included in the safety analysis set (22 in the DTx group and 23 in the control group). The full analysis set comprised 43 participants (20 in the DTx group and 23 in the control group) (Figure 2). There were no statistically significant differences between the groups in terms of baseline demographic characteristics or clinical features, except for sacral slope (SS) (Table 1). The sacral slope was significantly higher in the control group than that in the DTx group (38.6° vs. 34.0°, *p* = 0.035). The mean age was 38.1 ± 10.0 years in the DTx group and 38.5 ± 7.4 years in the control group (*p* = 0.930). Most of the participants were female (80.0% in the DTx group and 81.8% in the control group, *p* = 0.594). For baseline usual pain intensity (NPRS), the DTx group reported a mean score of 4.3 ± 1.4, while the control group reported 4.3 ± 1.3 (*p* = 0.910). For worst pain intensity (NPRS), the DTx group reported a mean score of 6.0 ± 1.6, while the control group reported 6.0 ± 1.7 (*p* = 0.990). For baseline functional disability measured by the ODI, the DTx group had a mean score of 19.7 ± 8.3, while the control group scored 19.6 ± 8.7 (*p* = 0.949)

### 3.2. Primary Outcomes

At eight weeks, no statistically significant differences in usual pain intensity were observed between the intervention and control groups in usual pain intensity (NPRS). The DTx group reported a mean score of 3.1 ± 1.9, while the control group reported 3.0 ± 1.5 (*p* = 0.809). Similarly, no significant differences were found in terms of worst pain intensity (NPRS) or ODI scores. The mean worst pain score was 5.00 ± 2.18 in the DTx group and 4.27 ± 1.83 in the control group (*p* = 0.247). The mean ODI score was 15.6 ± 9.6 for the DTx group and 15.6 ± 10.0 for the control group (*p* > 0.999).

### 3.3. Secondary Outcomes

**Figure 3:**
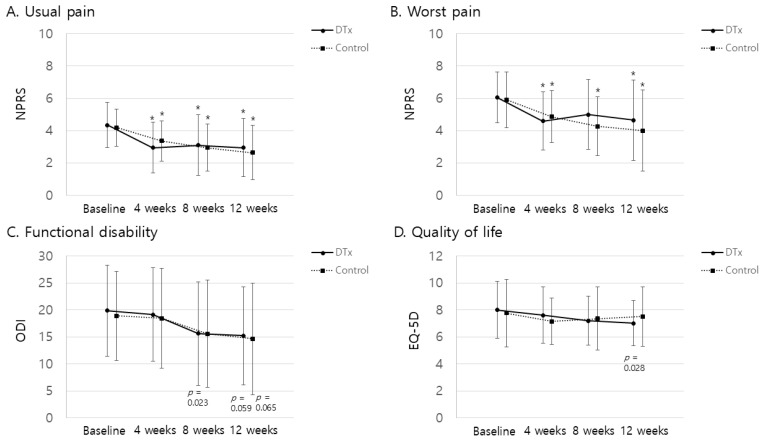
Changes in pain intensity, functional disability, and quality of life over time in the DTx and control groups. (**A**) Usual pain intensity measured by the Numeric Pain Rating Scale (NPRS) at baseline, 4 weeks, 8 weeks, and 12 weeks. (**B**) Worst pain intensity measured by NPRS over the same time points. (**C**) Functional disability, assessed by the Oswestry Disability Index (ODI), at baseline, 4 weeks, 8 weeks, and 12 weeks. (**D**) Quality of life, evaluated using the EQ-5D, at the same time intervals. * *p* < 0.05/3 compared with baseline.

**Figure 4:**
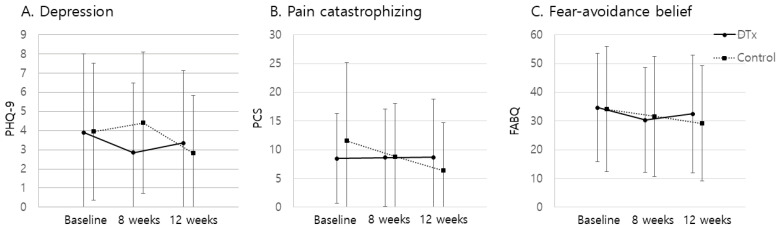
Changes in psychological outcomes over time in the DTx and control groups. (**A**) Depression levels measured by the Patient Health Questionnaire-9 (PHQ-9) at baseline, 8 weeks, and 12 weeks. (**B**) Pain catastrophizing, assessed by the Pain Catastrophizing Scale (PCS), at the same time points. (**C**) Fear-avoidance beliefs, measured by the Fear-Avoidance Beliefs Questionnaire (FABQ), over the same period. * *p* < 0.05/3 compared with baseline.

Both the DTx and control groups showed significant reductions in usual and worst pain over time compared with baseline (Figure 3). In the DTx group, the usual pain decreased significantly from baseline at 4, 8, and 12 weeks (*p* < 0.0167). Worst pain showed significant reductions at 4 and 12 weeks compared to baseline. In the control group, the usual and worst pain (NRS) scores significantly decrease at 4, 8, and 12 weeks. Although not statistically significant, improvements in ODI and quality of life EQ-5D were observed in both the DTx and control groups over time. No significant differences in secondary outcomes were observed between the groups at 4, 8, or 12 weeks. At 12 weeks, the mean usual pain intensity showed no significant difference, with the DTx group reporting 2.9 ± 1.8 compared to 2.6 ± 1.7 in the control group (*p* = 0.653). For worst pain intensity, there was also no significant difference, with scores of 4.7 ± 2.5 in the DTx group and 4.0 ± 2.5 in the control group (*p* = 0.406). Functional disability, measured by the ODI, showed no significant difference at 12 weeks, with the DTx group scoring 15.2 ± 9.2 and the control group scoring 14.6 ± 10.3 (*p* = 0.821). Similarly, quality of life, as measured by the EQ-5D, yielded no significant differences between the groups at any time point; at 12 weeks, the DTx group scored 7.0 ± 1.7, compared to 7.5 ± 2.2 for the control group (*p* = 0.610). Psychological measures, including depression (PHQ-9), pain catastrophizing (PCS), and fear-avoidance beliefs (FABQ), showed no significant differences between the groups at 8 or 12 weeks. Specifically, the PHQ-9 scores at 12 weeks were 3.4 ± 3.8 for the DTx group and 2.8 ± 3.0 for the control group (*p* = 0.704); the PCS scores were 8.7 ± 10.1 and 6.4 ± 8.4, respectively (*p* = 0.482); and the FABQ scores were 32.5 ± 20.4 and 29.4 ± 20.2, respectively (*p* = 0.689). Both the DTx and control groups showed no significant improvements in the PHQ-9, PCS, or FABQ scores compared with baseline at 8 or 12 weeks (Figure 4). The radiographic spinal alignment parameters generally showed no significant differences between the groups, except for the SS measured by the whole spine standing lateral radiograph at eight weeks, where the DTx group recorded 34.0 ± 8.0° compared to 38.6 ± 6.1° in the control group, indicating a significant difference (*p* = 0.035). Finally, no significant differences were observed between the groups in the Prone Bridge Test or Single Limb Stance Test at any time point. Between-group comparisons revealed a statistically significant difference in treatment compliance during the 1–4-week period (*p* < 0.001), with the DTx group showing a mean compliance of 74.7% ± 27.4, compared to 53.1% ± 28.6 in the control group. However, no significant differences were observed from 1 to 8 weeks (*p* = 0.145), as the intervention and control groups reported 64.7% ± 34.6 and 61.5% ± 29.2, respectively.

### 3.4. Safety Outcomes

The incidence of adverse events was similar between the intervention and control groups, with 77.27% (17/22) of the participants in the DTx group and 60.87% (14/23) in the control group experiencing at least one adverse event (*p* = 0.2348). The majority of adverse events were unexpected adverse device effects, whereas device-related adverse events (ADEs) were relatively infrequent, occurring in 18.18% (4/22) of the patients in the DTx group and none in the control group (*p* = 0.0490). The specific ADEs reported in the DTx group included musculoskeletal disorders (three participants, 13.64%), arthralgia (two participants, 9.09%), back pain (two participants, 9.09%), and neck pain (one participant, 4.55%). No serious adverse events were reported in the DTx group, and no unexpected device-related adverse events were reported in either group. Overall, the safety profiles were similar between the two groups, with most adverse events being mild to moderate in severity. No major safety concerns were observed in either group, suggesting that the MORA Cure LBP digital therapeutic device was generally well tolerated.

## 4. Discussion

This randomized controlled pilot study showed significant reductions in usual pain intensity, worst pain intensity, and functional disability over time with both multidisciplinary digital therapeutics (MORA Cure LBP) and conventional treatment groups. No statistically significant differences were observed between the two interventions for CLBP across various outcome measures. Although a statistically significant difference in sacral slope was noted between the groups (intervention group: 34.0°; control group: 38.6°; *p* = 0.035), the 4.6° difference likely falls within the range of measurement error and is unlikely to have clinical significance. Both treatments were safe and well tolerated, with MORA curing LBP demonstrating no serious adverse events and a safety profile comparable to that of conventional treatments.

When compared to similar studies, our results align with several previous findings in which digital therapeutics demonstrated improvements in pain management over time, although often with no significant differences between groups. Toelle et al. also found significant pain reduction using the Kaia App for back pain management, with no substantial differences between the digital and conventional therapy groups [28]. Similarly, Shi et al. demonstrated the non-inferiority of telerehabilitation compared to outpatient-based exercise, with both groups improving in terms of pain and disability [29]. Rughani et al. and Shebib et al. observed significant reductions in pain outcomes in both app-based interventions and control groups receiving conventional care [30,31]. In a study of 93 patients with low-back-pain-related disabilities, Chhabra et al. found that recovery rates were higher after 12 weeks of smartphone-based treatment (54.79%) than those after conventional treatment (51.52%) [32]. Almhdawi et al. also reported greater improvement in back-pain-related disability after six weeks of app-based intervention (35.70%) than that after conventional intervention (2.86%) in a study of 40 office workers with CLBP [33]. In our study, both the DTx and control groups showed improvements. The lack of a significant difference between the two groups may be due to the greater intensive care provided to the control group compared to that in previous studies. Unlike studies where the control group received only medication or verbal encouragement to exercise, our control group underwent monthly one-on-one supervised exercise sessions, up to four times in eight weeks, each time for >30 min, guided by an orthopedic surgeon or rehabilitation medicine specialist, which likely provided more comprehensive care.

Most other studies focused on exercise-based digital interventions without a significant CBT component. Our study included a comprehensive CBT component therapy combined with a structured exercise regimen. Shebib et al. demonstrated that a 12-week digital care program (DCP) combining CBT, exercise, and education significantly reduced pain intensity and functional disability in patients with CLBP compared to a control group receiving only educational materials [30]. Pain improved by 52–64%, and disability by 31–55% in the DCP group, with high weekly engagement (90%) and reduced surgical interest. Similarly, Chiauzzi et al. employed a self-management program featuring CBT alongside goal setting and wellness activities, demonstrating a significant improvement in disability outcomes but not in pain intensity [34].

Traditional CBT and exercise therapy, when conducted in regular face-to-face sessions, often pose financial burdens and constraints related to the time and location of patients. Psychological interventions conducted in humans have the same limitations. Enhancing the physical and time-related barriers, it is essential to overcome the accessibility of CBT and time-related barriers. Application-based (app-based) treatment is an excellent solution for addressing these challenges. Many studies have shown that the use of app-based interventions improves patient compliance [30,31,32,35,36,37,38]. In our study, the DTx group demonstrated a mean compliance of 74.7%, which was significantly higher than that of the control group (53.1% during the 1–4-week period (*p* < 0.001)). Shebib et al. reported a high weekly engagement rate of 90% in their digital care program [30]. Our study expressed engagement as an hour of rehabilitation, whereas Shebib’s study expressed the percentage of patients who completed treatment without dropping off for a certain period of time, making a direct comparison difficult; however, both studies showed good engagement. In a survey conducted with 127 healthcare professionals specializing in musculoskeletal disorders, 95.3% acknowledged the need for app-based treatment in clinical practice, and the majority expressed positive views toward its use [39]. Thus, integrating digital therapeutics for exercise and CBT could help improve treatment accessibility, reduce healthcare costs, and minimize inconvenience in patients with CLBP.

In our study, initial compliance to the intervention showed significant differences between the DTx and control groups during the first four weeks (*p* < 0.001), with DTx participants showing higher compliance (74.7% vs. 53.1%). However, from 1 to 8 weeks, the compliance rates were similar in both groups (64.7% vs. 61.5, *p* = 0.145). To optimize compliance and increase patient engagement, it is essential to explore the methods used in similar studies. For example, several app-based interventions aimed at chronic CLBP reported that enhancing user engagement through gamification and personalized feedback significantly improved adherence [30,32]. In a study by Shebib et al., the use of biofeedback and peer support within the app led to adherence rates as high as 90% in the first four weeks, although this decreased slightly over time [30]. In our study, the incorporation of CBT alongside exercise therapy was unique and could positively influence compliance; however, further improvement strategies, such as adding reward systems and more interactive features, might be beneficial [32]. Implementing real-time progress tracking, regular reminders, and personalized milestones could help maintain engagement to further increase compliance. Moreover, enhancing the usability of the application and ensuring that exercise routines are adaptable to the patient’s daily life are crucial steps for minimizing dropout rates [32]. By focusing on these aspects, we could potentially achieve higher adherence rates, as observed in other digital therapeutic studies.

Although no statistically significant differences were observed in secondary psychological outcomes (e.g., PHQ-9, PCS, FABQ) between the intervention and control groups, it is noteworthy that these measures also did not show significant improvement within either group at 8 or 12 weeks. This was unexpected, particularly given the inclusion of CBT in the intervention group. However, significant reductions in usual pain, worst pain, and functional disability were observed in both groups, suggesting that the mechanisms driving pain reduction may not be directly linked to changes in psychological measures. Further research is needed to elucidate the relationship between improvements in pain and functional outcomes and psychological measures, potentially identifying mediators that could enhance the effectiveness of digital therapeutics

The safety profile of the DTx group was comparable with that of the control group, and no significant safety concerns were observed throughout this study. Although the incidence of adverse events was slightly higher in the DTx group (77.27%) than that in the control group (60.87%), the difference was not statistically significant (*p* = 0.235). Most adverse events were mild or moderate in nature, and device-related adverse events were relatively infrequent (18.18% in the DTx group). These findings align with those of similar studies on digital therapeutics for chronic pain management, where adverse events were often mild and device-related incidents were infrequent. For example, a study involving AI-based self-management applications for musculoskeletal conditions reported a low incidence of adverse events, mostly attributable to normal fluctuations in pain during treatment [30,40]. In our study, arthralgia, back pain, and neck pain were the most commonly reported adverse events in the DTx group. Similar safety profiles were observed in related trials, such as those by Hartmann et al. and Shebib et al., who documented mild adverse events without serious risks associated with app-based therapies [30,41].

In our study, both groups showed significant pain improvements from the 4-week mark, consistent with previous studies indicating that chronic low back pain treatments yield benefits within the first month [42]. Although this pilot study demonstrates promising improvements in adherence and accessibility through digital therapeutics, the long-term efficacy and clinical relevance of such interventions remain to be fully established. Sustained adherence to digital platforms is likely to play a pivotal role in maintaining therapeutic benefits over time [43]. Evidence from prior studies indicates that higher initial compliance is often associated with better long-term outcomes in pain and disability management [44]. However, future large-scale, longitudinal studies are necessary to confirm whether these short-term benefits translate into lasting clinical improvements.

Our study has several limitations. First, the relatively short follow-up period restricts our ability to assess the long-term effects of the intervention. Second, although compliance was generally good, adherence tracking was not as detailed as that in the control group, limiting our understanding of comparative adherence. Third, the sample size was small, which may have affected the generalizability of our findings. Fourth, although our intervention combined CBT and exercise, we could not isolate the specific components that contributed the most to the observed outcomes. Future studies could employ a factorial design, including separate groups for CBT-only, exercise-only, and combined interventions, to better understand the individual and synergistic effects of these components. Such an approach would help refine the intervention and optimize its efficacy.

## 5. Conclusions

This pilot study demonstrated that multidisciplinary digital therapeutics (MORA Cure LBP) are safe and effective options for managing CLBP, resulting in significant improvements in pain, disability, and quality of life over time. The digital and conventional treatment groups achieved similar outcomes, with higher early compliance in the MORA Cure LBP group.

## Figures and Tables

**Table 1:** Baseline characteristics of study participants (full analysis set).

	Intervention (n = 20)	Control (n = 23)	*p* Value
Age (years)	38.1 ± 10.0	38.5 ± 7.4	0.930
Female (n [%])	16 [80.0]	18 [81.8]	0.594
Body mass index (kg/m^2^)	22.6 ± 4.4	23.4 ± 3.3	0.399
Prior pain medication use (n [%])	5 [25.0]	4 [17.4]	0.711
Onset of backpain (months)	88.9 ± 55.5	58.7 ± 52.6	0.078
Usual pain (NPRS)	4.4 ± 1.4	4.2 ± 1.1	0.792
Worst pain (NPRS)	6.1 ± 1.6	5.9 ± 1.7	0.679
Functional disability (ODI)	19.9 ± 8.4	18.9 ± 8.3	0.724
QoL (EQ-5D)	8.0 ± 2.2	7.8 ± 2.5	0.600
Depression (PHQ-9)	3.9 ± 4.1	3.8 ± 3.6	0.961
Pain catastrophizing (PCS)	8.5 ± 7.8	11.9 ± 13.4	0.654
Fear-avoidance beliefs (FABQ)	32.5 ± 20.4	29.4 ± 20.2	0.689
Muscle endurance (Prone Bridge, s)	63.2 ± 39.2	46.9 ± 32.6	0.161
Balance ability (Single Limb Stance)	4.3 ± 2.0	4.3 ± 2.4	0.897
Pelvic incidence (°)	48.3 ± 9.5	49.5 ± 7.1	0.649
Lumbar lordosis (°)	46.3 ± 12.3	49.8 ± 10.5	0.335
Sacral slope (°)	34.0 ± 8.0	38.6 ± 6.1	0.035
Pelvic tilt (°)	14.2 ± 8.5	10.9 ± 6.8	0.150
Sagittal vertical axis (mm)	5.3 ± 29.6	−3.7 ± 23.6	0.265
Cobb’s angle (°)	3.3 ± 2.3	2.8 ± 2.6	0.350

NPRS, Numeric Pain Rating Scale; ODI, Oswestry Disability Index; QoL, quality of life.

## Data Availability

Data underlying this article cannot be shared publicly because of the privacy of individuals who participated in the study. The data may be shared with the corresponding authors upon reasonable request.

## References

[1] van Tulder M., Becker A., Bekkering T., Breen A., del Real M.T.G., Hutchinson A., Koes B., Laerum E., Malmivaara A., COST B13 Working Group on Guidelines for the Management of Acute Low Back Pain in Primary Care (2006). European guidelines for the management of acute nonspecific low back pain in primary care. Eur. Spine J..

[2] van Dieën J.H., Kuijer P.P., Burdorf A., Marras W.S., Adams M.A. (2012). Non-specific low back pain. Lancet.

[3] Hartvigsen J., Hancock M.J., Kongsted A., Louw Q., Ferreira M.L., Genevay S., Hoy D., Karppinen J., Pransky G., Sieper J. (2018). What low back pain is and why we need to pay attention. Lancet.

[4] Foster N.E., Anema J.R., Cherkin D., Chou R., Cohen S.P., Gross D.P., Ferreira P.H., Fritz J.M., Koes B.W., Peul W. (2018). Prevention and treatment of low back pain: Evidence, challenges, and promising directions. Lancet.

[5] Abbafati C., Abbas K.M., Abbasi M., Abbasifard M., Abbasi-Kangevari M., Abbastabar H., Abd-Allah F., Abdelalim A., Abdollahi M., Abdollahpour I. (2020). Global burden of 369 diseases and injuries in 204 countries and territories, 1990–2019: A systematic analysis for the Global Burden of Disease Study 2019. Lancet.

[6] Maher C., Underwood M., Buchbinder R. (2017). Non-specific low back pain. Lancet.

[7] Costa L.D.M., Henschke N., Maher C.G., Refshauge K.M., Herbert D.R., McAuley J.H., Das A., Costa L.O.P. (2007). Prognosis of chronic low back pain: Design of an inception cohort study. BMC Musculoskelet. Disord..

[8] Hayden J.A., Chou R., Hogg-Johnson S., Bombardier C. (2009). Systematic reviews of low back pain prognosis had variable methods and results: Guidance for future prognosis reviews. J. Clin. Epidemiol..

[9] Hong J.Y., Song K.S., Cho J.H., Lee J.H., Kim N.H. (2022). An updated overview of low back pain management. Asian Spine J..

[10] Perrot S., Doane M.J., Jaffe D.H., Dragon E., Abraham L., Viktrup L., Bushmakin A.G., Cappelleri J.C., Conaghan P.G. (2022). Burden of chronic low back pain: Association with pain severity and prescription medication use in five large European countries. Pain Pract..

[11] Baroncini A., Maffulli N., Schäfer L., Manocchio N., Bossa M., Foti C., Klimuch A., Migliorini F. (2024). Physiotherapeutic and non-conventional approaches in patients with chronic low-back pain: A level I Bayesian network meta-analysis. Sci. Rep..

[12] Kuijpers T., van Middelkoop M., Rubinstein S.M., Ostelo R., Verhagen A., Koes B.W., van Tulder M.W. (2011). A systematic review on the effectiveness of pharmacological interventions for chronic non-specific low-back pain. Eur. Spine J..

[13] Kreiner D.S., Matz P., Bono C.M., Cho C.H., Easa J.E., Ghiselli G., Ghogawala Z., Reitman C.A., Resnick D.K., Watters W.C. (2020). Guideline summary review: An evidence -based clinical guideline for the diagnosis and treatment of low back pain. Spine J..

[14] O’Sullivan K., O’Keeffe M., O’Sullivan P. (2017). NICE low back pain guidelines: Opportunities and obstacles to change practice. Br. J. Sports Med..

[15] Nalamachu S., Rauck R.L., Hale M.E., Florete O.G., Robinson C.Y., Farr S.J. (2014). A long-term, open-label safety study of single-entity hydrocodone bitartrate extended release for the treatment of moderate to severe chronic pain. J. Pain Res..

[16] Kumar S., Negi M.P.S., Sharma V.P., Shukla R., Dev R., Mishra U.K. (2009). Efficacy of two multimodal treatments on physical strength of occupationally subgrouped male with low back pain. J. Back Musculoskelet. Rehabil..

[17] Ferreira M.L., Ferreira P.H., Latimer J., Herbert R.D., Hodges P.W., Jennings M.D., Maher C.G., Refshauge K.M. (2007). Comparison of general exercise, motor control exercise and spinal manipulative therapy for chronic low back pain: A randomized trial. Pain.

[18] Koldaş Doğan S., Sonel Tur B., Kurtaiş Y., Atay M.B. (2008). Comparison of three different approaches in the treatment of chronic low back pain. Clin. Rheumatol..

[19] Clarke J., van Tulder M., Blomberg S., de Vet H., van der Heijden G., Bronfort G. (2006). Traction for low back pain with or without sciatica: An updated systematic review within the framework of the Cochrane Collaboration. Spine.

[20] Ho E., Ferreira M., Chen L.X., Simic M., Ashton-James C., Comachio J., Hayden J., Ferreira P., Wang D.X.M., Ferreira P.H. (2022). Psychological interventions for chronic, non-specific low back pain: Systematic review with network meta-analysis. Br. Med. J..

[21] Fairbank J.C., Pynsent P.B. (2000). The Oswestry disability index. Spine.

[22] Rabin R., de Charro F. (2001). EQ-5D: A measure of health status from the EuroQol Group. Ann. Med..

[23] Kroenke K., Spitzer R.L., Williams J.B. (2001). The PHQ-9: Validity of a brief depression severity measure. J. Gen. Intern. Med..

[24] Darnall B.D., Sturgeon J.A., Cook K.F., Taub C.J., Roy A., Burns J.W., Sullivan M., Mackey S.C. (2017). Development and validation of a daily pain catastrophizing scale. J. Pain.

[25] Waddell G., Newton M., Henderson I., Somerville D., Main C.J. (1993). A Fear-Avoidance Beliefs Questionnaire (FABQ) and the role of fear-avoidance beliefs in chronic low back pain and disability. Pain.

[26] Bohannon R.W., Steffl M., Glenney S.S., Green M., Cashwell L., Prajerova K., Bunn J. (2018). The prone bridge test: Performance, validity, and reliability among older and younger adults. J. Bodyw. Mov. Ther..

[27] Balogun J.A., Ajayi L.O., Alawale F. (1997). Determinants of single limb stance balance performance. Afr. J. Med. Med. Sci..

[28] Toelle T.R., Utpadel-Fischler D.A., Haas K.K., Priebe J.A. (2019). App-based multidisciplinary back pain treatment versus combined physiotherapy plus online education: A randomized controlled trial. npj Digit. Med..

[29] Shi W., Zhang Y., Bian Y., Chen L., Yuan W., Zhang H., Feng Q., Zhang H., Liu D., Lin Y. (2024). The physical and psychological effects of telerehabilitation-based exercise for patients with nonspecific low back pain: Prospective randomized controlled trial. JMIR mHealth uHealth.

[30] Shebib R., Bailey J.F., Smittenaar P., Perez D.A., Mecklenburg G., Hunter S. (2019). Randomized controlled trial of a 12-week digital care program in improving low back pain. npj Digit. Med..

[31] Rughani G., Nilsen T.I.L., Wood K., Mair F.S., Hartvigsen J., Mork P.J., Nicholl B.I. (2023). The selfBACK artificial intelligence-based smartphone app can improve low back pain outcome even in patients with high levels of depression or stress. Eur. J. Pain.

[32] Chhabra H.S., Sharma S., Verma S. (2018). Smartphone app in self-management of chronic low back pain: A randomized controlled trial. Eur. Spine J..

[33] Almhdawi K.A., Obeidat D.S., Kanaan S.F., Oteir A.O., Mansour Z.M., Alrabbaei H. (2020). Efficacy of an innovative smartphone application for office workers with chronic non-specific low back pain: A pilot randomized controlled trial. Clin. Rehabil..

[34] Chiauzzi E., Pujol L.A., Wood M., Bond K., Black R., Yiu E., Zacharoff K. (2010). painACTION-back pain: A self-management Website for people with chronic back pain. Pain Med..

[35] Yang J.Y., Wei Q., Ge Y.L., Meng L.J., Zhao M.D. (2019). Smartphone-based remote self-management of chronic low back pain: A preliminary study. J. Healthc. Eng..

[36] Nordstoga A.L., Bach K., Sani S., Wiratunga N., Mork P.J., Villumsen M., Cooper K. (2020). Usability and acceptability of an app (SELFBACK) to support self-management of low back pain: Mixed methods study. JMIR Rehabil. Assist. Technol..

[37] Sitges C., Terrasa J.L., García-Dopico N., Segur-Ferrer J., Velasco-Roldán O., Crespí-Palmer J., González-Roldán A.M., Montoya P. (2022). An educational and exercise mobile phone-based intervention to elicit electrophysiological changes and to improve psychological functioning in adults with nonspecific chronic low back pain (BackFit app): Nonrandomized clinical trial. JMIR mHealth uHealth.

[38] Vad V.B., Madrazo-Ibarra A., Estrin D., Pollak J.P., Carroll K.M., Vojta D., Vad A., Trapness C. (2022). Back Rx, a personalized mobile phone application for discogenic chronic low back pain: A prospective pilot study. BMC Musculoskelet. Disord..

[39] Biebl J.T., Rykala M., Strobel M., Kaur Bollinger P.K., Ulm B., Kraft E., Huber S., Lorenz A. (2021). App-based feedback for rehabilitation exercise correction in patients with knee or hip osteoarthritis: Prospective cohort study. J. Med. Internet Res..

[40] Marcuzzi A., Nordstoga A.L., Bach K., Aasdahl L., Nilsen T.I.L., Bardal E.M., Boldermo N.Ø., Falkener Bertheussen G., Marchand G.H., Gismervik S. (2023). Effect of an Artificial Intelligence-Based Self-Management App on musculoskeletal Health in Patients with Neck and/or Low Back Pain Referred to Specialist Care: A Randomized Clinical Trial. JAMA Netw. Open.

[41] Hartmann R., Avermann F., Zalpour C., Griefahn A. (2023). Impact of an AI app-based exercise program for people with low back pain compared to standard care: A longitudinal cohort-study. Health Sci. Rep..

[42] Dillingham T., Kenia J., Popescu A., Plastaras C., Becker S., Shofer F. (2020). Pain outcomes with an elliptical regimen (POWER) study: Identifying the proper dosage of exercise for therapeutic effect in persons with chronic back pain. J. Phys. Med. Rehabil..

[43] Li Y., Gong Y., Zheng B., Fan F., Yi T., Zheng Y., He P., Fang J., Jia J., Zhu Q. (2022). Effects on adherence to a mobile app-based self-management digital therapeutics among patients with coronary heart disease: Pilot randomized controlled trial. JMIR mHealth uHealth.

[44] Areias A.C., Costa F., Janela D., Molinos M., Moulder R.G., Lains J., Scheer J.K., Bento V., Yanamadala V., Correia F.D. (2022). Long-term clinical outcomes of a remote digital musculoskeletal program: An ad hoc analysis from a longitudinal study with a non-participant comparison group. Healthcare.

